# ABC Transport Is Inactivated by the PTS^Ntr^ under Potassium Limitation in *Rhizobium leguminosarum* 3841

**DOI:** 10.1371/journal.pone.0064682

**Published:** 2013-05-28

**Authors:** Verena Untiet, Ramakrishnan Karunakaran, Maria Krämer, Philip Poole, Ursula Priefer, Jürgen Prell

**Affiliations:** 1 Soil Ecology, RWTH Aachen, Aachen, Germany; 2 Molecular Microbiology, John Innes Centre, Norwich, United Kingdom; University of Cambridge, United Kingdom

## Abstract

PTS^Ntr^ is a regulatory phosphotransferase system in many bacteria. Mutation of the PTS^Ntr^ enzymes causes pleiotropic growth phenotypes, dry colony morphology and a posttranslational inactivation of ABC transporters in *Rhizobium leguminosarum* 3841. The PTS^Ntr^ proteins EI^Ntr^ and 2 copies of EIIA^Ntr^ have been described previously. Here we identify the intermediate phosphocarrier protein NPr and show its phosphorylation by EI^Ntr^ *in vitro*. Furthermore we demonstrate that phosphorylation of EI^Ntr^ and NPr is required for ABC transport activation and that the N-terminal GAF domain of EI^Ntr^ is not required for autophosphorylation. Previous studies have shown that non-phosphorylated EIIA^Ntr^ is able to modulate the transcriptional activation of the high affinity potassium transporter KdpABC. In *R. leguminosarum* 3841 *kdpABC* expression strictly depends on EIIA^Ntr^. Here we demonstrate that under strong potassium limitation ABC transport is inactivated, presumably by non-phosphorylated EIIA^Ntr^. This is to our knowledge the first report where PTS^Ntr^ dictates an essential cellular function. This is achieved by the inverse regulation of two important ATP dependent transporter classes.

## Introduction

Rhizobia are proteobacteria that are best known for their ability to enter a nitrogen fixing symbiosis with legume plants. After an exchange of signaling compounds rhizobia enter the appropriate host plant root in general via plant derived infection threads [1]. These infection threads grow into the root cortex towards a newly induced meristem that forms the basis for a newly developing root structure called a nodule [2]. Inside the nodule the plant cells greatly enlarge and their cytosol fills up with infection thread derived symbiosomes [3]. Symbiosomes are surrounded by a plant derived membrane and contain rhizobia. Inside these symbiosomes rhizobia transform into nitrogen fixing bacteroids. Bacteroids are greatly dependent on plant derived compounds and are therefore considered as plant organelles, called ammoniaplasts [4]. Exchange of molecules requires transport across two membranes, the bacteroid- and the symbiosome membrane. Rhizobia undergo dramatic changes in their lifestyle by turning from an oligotrophic soil bacterium into an intracellular plant symbiont [5].

Rhizobia are also known to be especially rich in ATP binding cassette dependent transporters, so called ABC transporters [6]. ABC transporters are widely spread in all kingdoms of life and have high affinities for the solutes they transport. The *Rhizobium leguminosarum* 3841 genome sequence contains 269 genes encoding for ATP binding cassettes, which are characteristic for ABC transporters. Together with additional permease- and solute binding proteins, they are thought to make up more than 180 individual ABC transport systems that are largely uncharacterized. This high number of ABC systems probably reflects the requirements of a soil dwelling bacterium competing in a nutrient poor environment. In a systematic approach in *Sinorhizobium meliloti* 1021 it has been shown that a large fraction of the ABC transport systems can be specifically induced, presumably by the solutes they transport [6]. In *R. leguminosarum* 3841 it has also been demonstrated that a large number of ABC systems are generally expressed [7], [8]. Very recently it has been shown that ABC transport can be regulated post-transcriptionally [9], [10].

One of these regulatory circuits involves the PTS^Ntr^. Phospho-transferase systems (PTS) are known since more than 50 years. The first PTS was described in *E. coli* as a phosphorylation cascade that transfers phosphate from phosphoenolpyruvate (PEP) to incoming sugars [11]. PTS systems are made up of a core set of three enzyms: Enzyme I (EI), histidine protein (HPr) and Enzyme IIA (EIIA). EI autophosphorylates on a conserved histidine residue in the presence of PEP. This phosphate is then transferred to a conserved histidine on the phosphocarrier protein HPr, which finally phosphorylates the EIIA component, also on a conserved histidine residue. Multiple EIIA components in *E. coli* interact with multiple EIIBC(D) transport complexes which facilitate uptake and phosphorylation mainly of incoming sugar compounds.

More recently an EIIA homologue (PtsN) was discovered within the *rpoN* operon in *E. coli* [12] which also contains an HPr homologue named NPr [13]. A homologous EI component (EI^Ntr^ or PtsP) was later identified [14], which completed the paralogous PTS^Ntr^ system. PTS^Ntr^ does not interact with PTS transport complexes and was named after its presence in the *rpoN* operon and its probable involvement in regulation of nitrogen (Ntr) metabolism. The PTS^Ntr^ components are specifically named EI^Ntr^ (or PtsP), NPr (or PtsO) and EIIA^Ntr^ (or PtsN). EI^Ntr^ contains an N-terminal GAF domain of 180 aa which separates it from EI proteins of the transport PTS. PTS^Ntr^ is widely distributed in bacteria and it has been suggested that transport PTS components are rather an exception than the rule [15]. In *R. leguminosarum* 3841 and all other rhizobia sequenced so far PTS transport components are lacking but PTS^Ntr^ components are present [16].

The main functions of PTS^Ntr^ are still largely unknown, although mutants display pleiotropic phenotypes. In recent years, the *E. coli* PTS^Ntr^ has been linked to potassium homeostasis, because non-phosphorylated PtsN inhibits the low affinity K^+^ transporter Trk via protein-protein interaction with TrkA [17] and at the same time transcriptionally activates the high affinity K^+^ transporter KdpFABC via protein-protein interaction with KdpD, a sensor kinase that acts via the response regulator KdpE [18]. Additionally, non-phosphorylated PtsN also activates the Pho regulon, responsible for phosphate acquisition, via protein-protein interaction with the master regulator PhoR in *E. coli* [19].

In other bacteria PTS^Ntr^ mutants have exhibited a wide range of diverse phenotypes [20]. These phenotypes are often related to carbon metabolism and protein-protein interactions with central metabolic enzymes have been described very recently [21], [22]. PTS^Ntr^ mutants were also investigated in rhizobia. In *Sinorhizobium meliloti* 1021 PTS mutants were linked to succinate mediated catabolite repression (SMCR) [23], [24], in *R. etli* a *ptsN* mutant showed reduced growth on dicarboxylates, reduced melanin production and *nifH* induction [25] and in *Bradyrhizobium japonicum* I110 a *ptsP* mutant showed strongly reduced oligopeptide uptake [26].

We have recently demonstrated that PtsP and two copies of PtsN are required for the full activation of a wide range of ABC transporters in *R. leguminosarum* 3841 [10]. We also showed that PtsN, presumably in its non-phosphorylated form, interacts with KdpD, the sensor kinase that transcriptionally activates the high affinity K^+^ transporter KdpABC via the response regulator KdpE, identically to the situation in *E. coli*.

Here we identify the missing phosphocarrier protein NPr of the PTS^Ntr^ in Rlv3841 and characterise critical components of the phosphorylation cascade. We also demonstrate that under potassium limitation the phosphorylation state of PTS^Ntr^ dictates the activity of two essential ATP dependent transporter classes. This is to our knowledge the first report of an essential regulation mediated by PTS^Ntr^.

## Results

### NPr Candidates in *Rhizobium leguminosarum* 3841

We have recently described the PTS^Ntr^ phosphorylation cascade in *R. leguminosarum* 3841 and its role in the activation of ABC transport and regulation of high affinity K^+^ uptake [10]. EI^Ntr^ and two copies of EIIA^Ntr^ are part of this phospho relay and linked by phenotypes, but the intermediate NPr has not been identified. An inspection of the Rlv3841 genome produced two possible candidate genes, RL0032 and RL2903, which are annotated as putative phosphocarrier proteins. RL0032 shows 37% and 33% identity to NPr and HPr from *E. coli* K12, while RL2903 has 28% and 31% identity, respectively. RL0032 is the last gene in a possible operon that is conserved in many bacteria [24], [35], [36] and involved in SMCR in *Sinorhizobium meliloti* 1021 [23]. Upstream of RL0032 is a gene coding for a protein with homology to the N-terminus of EIIA^Man^, a mannose PTS component, and further up-stream is a gene coding for a putative HPr kinase (HprK). Mutants in those genes in *S. meliloti* 1021 are all linked to SMCR and carbon metabolism, but also to cobalt requirements, succinoglycan production and symbiotic performance [23], [24]. The authors describe a possible PTS in *S. meliloti* starting with the phosphorylation of histidine 22 of HPr by EI^Ntr^. The final phosphorylation acceptor of this cascade is probably EIIA^Man^. The second *npr* candidate, RL2903, is part of a dihydroxy acetone PTS which is likely functional in rhizobia [37].

### RL0032 is NPr, the Missing link in the PTS^Ntr^ Phosphorylation Cascade

We mutated RL0032 by replacing the gene with an ΩTet marker cassette creating strain AA015 (ΔRL0032). AA015 grew well on TY medium and displayed a dry colony morphology identical to PtsP107 (*ptsP*::Tn*5*). We also replaced RL2903, the second phosphocarrier protein candidate gene, with an ΩSpec cassette, creating strain AA006 (ΔRL2903) and the double mutant AA016 (ΔRL0032; ΔRL2903). Strain AA006 formed mucoid colonies comparable to wild type Rlv3841 and AA016 formed dry colonies identical to AA015 (ΔRL0032) and PtsP107 (*ptsP*::Tn*5*). This clearly links RL0032 by phenotype to PTS^Ntr^ mutants and we therefore tentatively named RL0032: NPr and the AA015 (ΔRL0032) mutant: AA015 (Δ*npr*).

**Figure 1:**
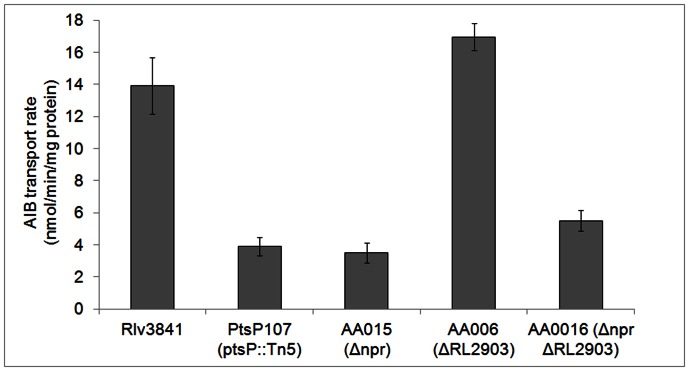
AIB membrane transport by Rlv3841, PtsP107 (ptsP::Tn5) and various phosphocarrier protein mutants. Data are averages (± SEM) from 6 independent cultures and rates are nmol min^−1^mg protein^−1^.

Because EI^Ntr^ (PtsP) and EIIA^Ntr^ (PtsN1/2) mutants (PtsP107 and LMB272; [10]) display similar colony morphology phenotypes which are linked to ABC transporter activities, we measured the uptake activities of the 2 general amino acid ABC transporters Aap and Bra in Rlv3841, PtsP107 (*ptsP*::Tn*5*) and the different phosphocarrier protein mutants, AA015 (Δ*npr*), AA006 (ΔRL2903) and AA016 (Δ*npr*; ΔRL2903). Figure 1 shows α-amino isobutyric acid (AIB; a non metabolisable amino acid analog) uptake rates of the different strains grown in AMS with glucose and ammonium. Rlv3841 transported AIB with a typical rate of ∼14 nmol min^−1^ mg protein^−1^, while transport rates of PtsP107 (*ptsP*::Tn*5*), AA015 (Δ*npr*) and AA016 (Δ*npr*; ΔRL2903) were reduced by ∼75%. AA006 (ΔRL2903) showed wild type rates of AIB transport. Glucose is also transported via at least 3 different ABC transport systems in Rlv3841 [7] and transport rates were reduced by ∼50% in PtsP107 [10]. Glucose transport rates of AA015 (Δ*npr*) and AA016 (Δ*npr*; ΔRL2903) were also reduced by ∼50% compared to Rlv3841 and AA006 (ΔRL2903) (data not shown).

The colony morphology and ABC transport phenotypes of the *npr* (RL0032) mutant AA015 are very similar to the phenotypes of the other PTS^Ntr^ mutants, which suggests that NPr is the missing phosphocarrier protein which should be phosphorylatable by EI^Ntr^ (PtsP).

### EI^Ntr^ (PtsP) Autophosphorylates with PEP and Phosphorylates NPr *in vitro*

To test the hypothesis that EI^Ntr^ (PtsP) can phosphorylate NPr *in vitro* we purified His-tagged versions of both proteins. 6His-PtsP and 6His-GST-PtsP fusions were not soluble in *E. coli* BL21 DE3+ cells in sufficient amounts, but 6His-MBP-PtsP (130 kD) and 6His-NPr (10 kD) could be purified using Ni-IDA affinity purification. Both purified proteins showed only very minor contaminations.

**Figure 2:**
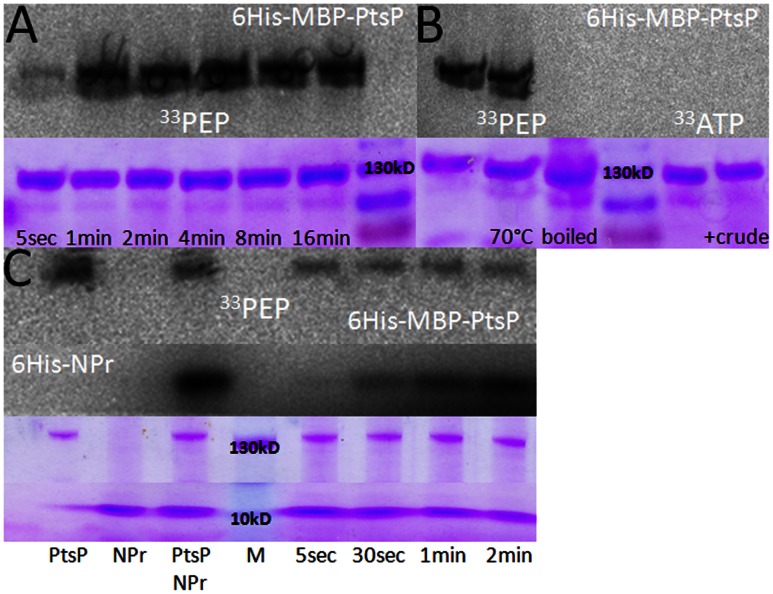
In vitro phosphorylation of purified 6His-MBP-PtsP protein. (A) Example of a time course of 6His-MBP-PtsP phosphorylation. Each lane of the SDS-page contains ∼2 µg of protein and was incubated with 125 µM [^33^P]PEP. The upper part shows the level of phosphorylation and the lower part the same gel after coomassie blue stain. (B) Phosphorylation of 6His-MPB-PtsP under various conditions. The phosphorylation is stable at 70°C for 10 min and boiled protein is inactive. The protein is not phosphorylated with 10 mM [^33^P]ATP and also not in the presence of crude extract of RU4392 (Δask). (C) *In vitro* phosphorylation of purified 6His-MBP-PtsP and 6His-NPr protein. Lane 1–3 show phosphorylation of the single proteins and both proteins together with 125 µM [^33^P]PEP. Lane 5–8 show a timecourse of the transphosphorylation. The upper part shows the level of phosphorylation and the lower part the same gel after coomassie blue stain.

6His-MBP-PtsP rapidly autophosphorylated when ^33^PEP was added to the assay (Fig. 2A). This phosphorylation was heat stable at 70°C for 10 min and did not occur when 6His-MBP-PtsP was inactivated by boiling before the reaction start (Fig. 2B). The histidine phosphorylation of the smaller HPr protein of *Bacillus subtilis* is instable under those conditions [38]. Phosphorylation of 6His-MBP-PtsP did not occur when ^33^ATP was used (Fig. 2B). The addition of protein crude extract of the *Rlv*3841 asparto kinase mutant RU4392 (Δask; [10]) and ^33^ATP did also not result in phosphorylation, even though this was reported for purified GST-PtsP protein from *B. japonicum* I110 [26].

6His-NPr did not autophosphorylate upon addition of ^33^PEP (Fig. 2C, lane 2). When 6His-MBP-PtsP was first incubated with ^33^PEP for 15 min and 6His-NPr was added for 15 min, it became strongly phosphorylated (Fig. 2C, lane 3). When both proteins were mixed and incubated with ^33^PEP, 6His-MBP-PtsP rapidly autophosphorylated and the phosphate was then transferred to 6His-NPr (Fig. 2C, lanes 5–8).

This demonstrates that the NPr protein is phosphorylated by EI^Ntr^ (PtsP) *in vitro* and is therefore very likely part of the PTS^Ntr^ phosphorylation cascade.

### EI^Ntr^ (PtsP) Requires Histidine 367 for ABC Transport Activation, but Autophosphorylation is Probably not Dependent on its GAF Domain

**Figure 3:**
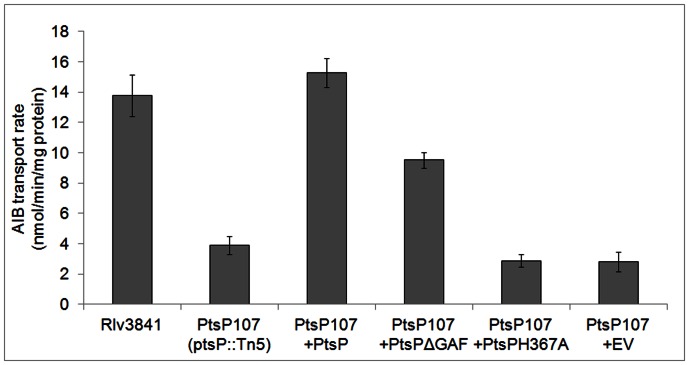
AIB transport by Rlv3841 and PtsP107 (ptsP::Tn5) and PtsP107 complemented with the empty vector pRK415 (EV) or PtsP, expressed from the constitutive lac promoter in multi-copy (+PtsP). Additionally, two variants, +PtsPH367A and +PtsPΔGAF, were used for complementation. Data are averages (± SEM) from ≥3 independent cultures and rates are nmol min^−1^ mg protein^−1^.

Since we demonstrated that EI^Ntr^ (PtsP) phosphorylates NPr *in vitro*, we wanted to test which functional properties of EI^Ntr^ (PtsP) are necessary to enable the phosphorylation cascade *in vivo*. EI^Ntr^ (PtsP) is a multi domain protein with an N-terminal GAF domain and several PEP utilizing domains, including a central domain that contains histidine 367, which is the conserved residue for autophosphorylation with PEP. GAF domains are well known as regulatory small ligand binding domains [20] and they are characteristic for EI^Ntr^ proteins in comparison to EI enzymes of PTS sugar transport systems [14]. The EI^Ntr^ GAF domain is thought to be the regulatory input domain of the phosphorylation cascade, but possible ligands are so far unknown [20]. We have previously shown that we can complement the *ptsP* mutant PtsP107 *in trans*, expressing *ptsP* from the *E. coli lacZ* promoter on a low copy plasmid pRK415 (pLMB151; [10]). To verify the role of the histidine 367 residue and the GAF domain we constructed two pRK415 derivatives expressing a PtsP with histidine 367 replaced by an alanine (pAA003; PtsPH367A) and a PtsP lacking the N-terminal GAF domain (pAA001; PtsPΔGAF). Both plasmids were introduced into the *ptsP* mutant PtsP107, forming strains PtsP107+PtsPH367A and PtsP107+PtsPΔGAF, and were compared to the fully complemented strain (Pts107+PtsP) and an empty vector control (PtsP107+EV). Colonies of Pts107+PtsP and PtsP107+PtsPΔGAF showed a surface phenotype identical to wild type Rlv3841. In contrast PtsP107+EV and PtsP107+PtsPH367A looked identical to PtsP107. To further analyse the changes in phenotype we assayed the complemented strains for AIB uptake (Fig. 3). Strains Rlv3841 and Pts107+PtsP showed comparable rates of AIB transport of ∼14 nmol min^−1^ mg protein^−1^, while PtsP107, PtsP107+EV and PtsP107+PtsPH367A showed severely reduced transport rates. Interestingly, we measured a small but significant reduction of AIB transport (p<0.001; n = 6) in the PtsP107+PtsPΔGAF compared to the fully complemented PtsP107+PtsP strain. Very similar results were obtained when glucose uptake was quantified (data not shown). These results demonstrate that the conserved phosphorylation site histidine 367 of EI^Ntr^ (PtsP) is necessary for ABC transport activation and that a GAF truncated version of PtsP is able to sufficiently activate the PTS^Ntr^ phosphorylation cascade and therefore ABC transport. To further clarify the role of the GAF domain, autophospho-rylation of purified GAF truncated PtsP protein needs to be tested *in vitro*.

### NPr Requires Histidine 17 for ABC Transport Activation

**Figure 4:**
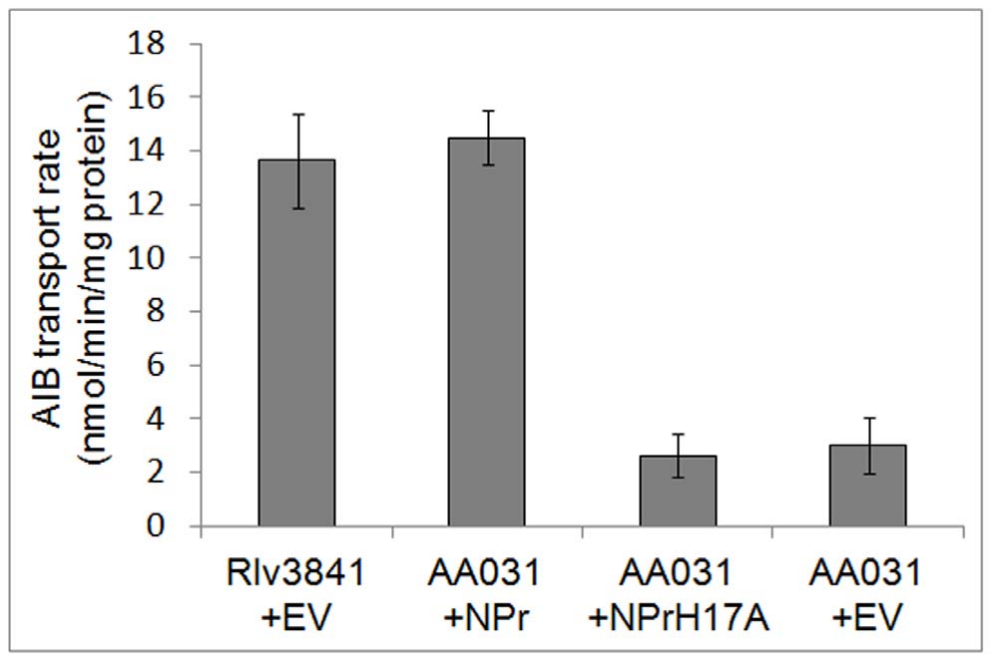
AIB transport by Rlv3841 and AA031 (Δ*npr*) and AA031 complemented with the empty vector pRK415 (EV), NPr, expressed from the constitutive lac promoter in multi-copy (+NPr) or a non-phosphorylatable variant (+NPrH17A). Data are averages (± SEM) from ≥3 independent cultures and rates are nmol min^−1^ mg protein^−1^.

Since phosphorylation of PtsP is essential for ABC transport activation, we also wanted to verify that this is true for NPr. NPr contains a conserved histidine at position 17 which is the proposed site of phosphorylation by PtsP. To test the influence of the histidine 17 phosphorylation site we cloned a wild type NPr and a NPrH17A variant into pRK415 to complement an NPr mutant in Rlv3841. For that we first had to replace the ΩTet marker of AA015 (Δ*npr*) by an ΩSpec marker resulting in strain AA031 (Δ*npr*). AA015 and AA031 exhibited indistinguishable surface and transport phenotypes (data not shown). AA031 (Δ*npr*) was complemented with NPr (pAA038) and NPrH17A (pAA039) and the empty vector (EV). Complementation with NPr restored the mucoid Rlv3841 surface phenotype, while NPrH17A and the EV produced dry colonies in the AA031 background. Additionally, AIB transport rates of Rlv3841+EV and AA031+NPr were at WT level of ∼14 nmol/min/mg protein (Fig. 4), while AA031+NPrH17A and AA031+EV transported with strongly reduced rates comparable to PtsP107+EV (compare to Fig. 3).

We therefore conclude that H17 phosphorylation of NPr is required for ABC transport activation.

### ABC Transport Activity is Reduced under K^+^ Limitation

It has previously been shown in *E. coli* that non-phosphorylated PtsN (EIIA^Ntr^) activates the ATP dependent high affinity K^+^ transporter KdpFABC (which is not an ABC transporter) via the stimulation of phosphorylation of the two component transcriptional regulator KdpDE [18]. We have recently demonstrated in *R. leguminosarum* 3841 that a *kdpA* mutant shows the same severe growth defects in medium with limiting K^+^ levels (≤10 µM KCl) as a *ptsN1/N2* double mutant, indicating that PtsN is essential for Kdp activation. In contrast, wild type and a *ptsP* mutant, which cannot phosphorylate PtsN, grow equally well down to levels of 1 µM KCl [10] indicating that non-phosphorylated PtsN is required for *kdpABC* expression and therefore activation. Consistent with that, we have shown that *kdpAB* expression is up regulated in a *ptsP* mutant and that PtsN1 and PtsN2 can both physically interact with KdpD. Altogether these results demonstrate that as in *E. coli* non-phosphorylated PtsN is required for full *kdpABC* transcription and high affinity K^+^ transport. However, in contrast to *E. coli*, where *kdpFABC* transcription is only modulated by PtsN dependent on an unknown signal, in *R. leguminosarum* 3841 K^+^ transport via KdpABC is completely dependent on the presence of PtsN, presumably in its non-phosphorylated form, at growth limiting K^+^ levels.

This allows us to make the following prediction: If non-phosphorylated PtsN is required for growth of wild type Rlv3841 at limiting K^+^ levels (≤10 µM KCl), ABC transport rates should be reduced under those conditions.

**Figure 5:**
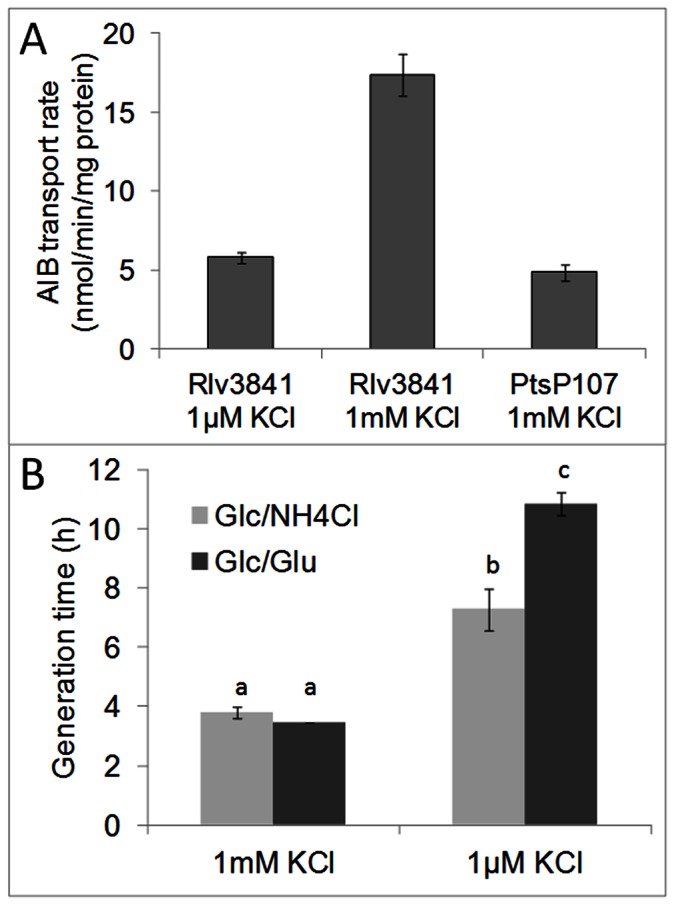
Effects of K^+^ limitation on ABC transport and growth of Rlv3841. (A) AIB transport by Rlv3841 at 1 µM and 1 mM KCl and PtsP107 as a control. Data are averages (± SEM) from 3 independent cultures and rates are nmol min^−1^ mg protein^−1^. (B) Generation times of Rlv3841 grown at 1 µM and 1 mM KCl with 10 mM NH_4_Cl or 10 mM glutamate as the sole nitrogen source. Data are averages (± SEM) from 3 independent experiments with each 2 cultures. Data indicated with different letters are significantly different in a Student’s t-test (p<0.01).

To test this, we grew Rlv3841 in AMS with normal and limiting K^+^ levels (1 mM and 1 µM KCl, resp.) and compared AIB transport rates to those of the *ptsP* mutant PtsP107 (Fig. 5A). Rlv3841 grown at 1 mM KCl transported AIB at normal rates (∼17 nmol/min/mg protein). At limiting K^+^ levels transport of K^+^ via KdpABC is essential for growth and its transcriptional activation is dependent on the presence of non-phosphorylated PtsN. When Rlv3841 was grown at 1 µM K^+^, AIB transport rates dropped to the levels present in a *ptsP* mutant (∼5 nmol/min/mg protein), where PtsN cannot be phosphorylated via EI^Ntr^ (PtsP) and Npr. This shows that under K^+^ limitation, ABC transport is inactivated to an extent similar to that in a *ptsP* mutant.

From that result we also expected that Rlv3841 would show difficulties to grow on AMS Glc/Glu medium when potassium is limiting, because glutamate transport is dependent on the ABC transport systems Aap and Bra, while NH_4_^+^ transport is independent from ATP dependent transport systems. Therefore we determined generation times of Rlv3841 grown on AMS Glc/NH_4_Cl compared to AMS Glc/Glu at non-limiting potassium levels (1 mM KCl) compared to limiting levels (1 µM KCl). Generation times of Rlv3841 are very similar (∼4h) when grown on AMS Glc/NH_4_Cl compared to AMS Glc/Glu at 1 mM KCl (Fig. 5B and [10]). When grown at strong potassium limitation (1 µM KCl) generation times markedly increased (Fig. 5B and [10]) and this increase was significantly stronger when grown in AMS Glc/Glu (p = 0.009; n = 3 independent experiments with each 2 independent cultures).

## Discussion

We have recently identified the EI^Ntr^ and two EIIA^Ntr^ proteins of the PTS^Ntr^ of *R. leguminosarum* 3841 [10]. EI^Ntr^ and EIIA^Ntr^ mutants exhibit similar dry surface phenotypes and showed reduced activities of a range of ABC transporters. We suspected that the dry surface is a result of reduced EPS secretion which is likely facilitated via a yet uncharacterised ABC exporter [10], [39]. Additionally it was demonstrated that EIIA^Ntr^, presumably in its non-phosphorylated form, is required for the activation of the high affinity potassium transporter KdpABC and therefore essential for growth on limiting potassium levels. The intermediate HPr homologue of the PTS^Ntr^ phosphorylation cascade was left unidentified.

Here we first identify the missing intermediate phosphocarrier protein NPr that connects the phosphorylation from EI^Ntr^ (PtsP) to EIIA^Ntr^ (PtsN). The *npr* gene (RL0032) is in *R. leguminosarum* 3841 not located within the *rpoN* operon together with *ptsN* (EIIA^Ntr^) as in many other bacterial genomes [20]. In contrast, *npr* is located within another conserved operon downstream of the two component regulator genes *chvI* and *chvG*, a putative *hprK* gene and a *manX* homologue which encodes a protein with homologies to EIIA^Man^ proteins. The conservation of this operon has been mentioned previously [24], [35], [36], and seems to be restricted to α-proteobacteria [35]. But it remains to be determined whether the absence of *npr* (usually named *ptsO*) within the *rpoN* operon is strictly linked to the presence of the *chvIG*, *hprK*, *manX*, *npr* operon. In *B. melitensis* it has been suggested and demonstrated by RT-PCR that this operon is co-transcribed from a single *chvI* promoter [21], although the operon structure in *R. leguminosarum* 3841 and related species allows sufficient space for a *manX* promoter downstream of *hprK.* The NPr homologue in *S. meliloti* 1021 is named HPr [23], [24].

The Rlv3841 *npr* mutants AA015 (*npr*::ΩTet) and AA031 (*npr*::ΩSpec) exhibit identical surface and transport phenotypes to the *ptsP*::Tn5 (PtsP107) mutant (Fig. 1 and 4). Additionally, purified 6His-MBP-PtsP was able to phosphorylate 6His-NPr *in vitro* (Fig. 2C). This transphosphorylation was also recently demonstrated for the PtsP and NPr homologues of *B. melitensis* [21]. In this study it was additionally shown that NPr is able to phosphorylate EIIA^Ntr^ as well as EIIA^Man^. We assume this is also likely the case for the homologous Rlv3841 and *S. meliloti* 1021 proteins, because a phenotypical link between NPr (called Hpr in the following citation) and EIIA^Man^ (ManX) was also made in Rm1021 [23]. In that study phenotypes of NPr and EIIA^Man^ mutants were linked to SMCR and carbon metabolism. In the next study of this group [24] HprK was also shown to be involved in the regulation of SMCR. The mechanism of this regulation is still largely unknown but it is mediated via a conserved serine residue of NPr, similar to the regulation of carbon catabolite repression in firmicutes [40]. This conserved serine (S48) is also present in the NPr of *R. leguminosarum* 3841and suggests a second level of PTS^Ntr^ regulation involving carbon metabolism. An interaction of EIIA^Ntr^ (PtsN) with pyruvate dehydrogenase has recently been demonstrated in *P. putida* [22] and an interaction of EIIA^Man^ (ManX) with α-ketoglutarate dehydrogenase is likely to occur in *B. melitensis* [21]. A general regulation of central carbon metabolism seems likely.

After the identification of NPr we show direct mechanistic evidence that phosphorylation of the PTS^Ntr^ proteins is required for ABC transport activation in Rlv3841. Non-phosphorylatable versions of *ptsP* (PtsPH367A) and *npr* (NPrH17A) are not able to activate AIB (and glucose) transport above the levels of the respective mutants (Fig. 3 and 4). It was crucial to demonstrate this because these results are in contrast to a previous result presented in Prell *et al*. 2012, where a non-phosphorylatable version of EIIA^Ntr^ (PtsN1H66A) was able to restore AIB transport almost to wild type levels in an EIIA^Ntr^ double mutant background (LMB272) when expressed from the *lacZ* promoter of the mid-copy number plasmid pBBR-MCS5. In this study we concluded that non-phosphorylated PtsN must be a weak activator of AIB transport and that a complementation needs to be repeated with reduced copy numbers of PtsN1H66A to make final conclusions. The level of PtsN protein and its relative degree of phosphorylation seems to be absolutely crucial for its function in a physiological context. This is in agreement with recent results in *E. coli* where non-phosphorylatable PtsN is required to activate PhoR [19]. However, in a physiological condition where *E. coli* PtsN is shown to be fully phosphorylated [41], both phosphorylatable and non-phosphorylatable PtsN versions were equally able to activate PhoR mediated transcriptional responses when expressed from a plasmid [19]. This clearly shows that absolute levels and relative degree of phosphorylation are essential for PtsN function.

After we show the importance of phosphorylation of the PTS^Ntr^ proteins we also demonstrate that the N-terminal EI^Ntr^ GAF domain is dispensable for ABC transport activation (Fig. 3). We suspect the GAF domain must have a negative regulatory function in the control of EI^Ntr^ phosphorylation, because its removal still allows significant ABC transport activation which is dependent on phosphorylation of the PTS^Ntr^ cascade. In a physiological context, *e.g.* under potassium limitation, when growth is dependent on *kdpABC* expression and PtsN should be predominantly non-phosphorylated, the cell must either be depleted in PEP levels or an unknown ligand must bind to the EI^Ntr^ GAF domain and prevent EI^Ntr^ and therefore NPr and EIIA^Ntr^ phosphorylation. We believe that PEP depletion is an unlikely condition, because growth on glucose is not limited in PTS^Ntr^ mutants [10]. We instead predict that a ligand, either building up or depleting under limiting K^+^ conditions, might control EI^Ntr^ autophosphorylation or NPr transphosphorylation. The nature of that ligand might reflect the adenylate charge of the cell as already discussed in Prell *et al*. 2012, because in *R. leguminosarum* two ATP dependent transporter classes, ABC transport and KdpABC, are both regulated by PTS^Ntr^. Such a ligand has so far not been identified [20], although a wide range of obvious candidates were tested with the *E. coli* EI^Ntr^ [42]. However, the situation in an α-proteobacterium might be different from *E. coli*.

Furthermore, if under conditions of potassium limitation EIIA^Ntr^ phosphorylation is strongly reduced, this should also result in reduced ABC transport activation. We could indeed demonstrate that AIB transport in Rlv3841 was reduced to *ptsP* mutant levels when potassium concentrations were at 1 µM in the growth medium (Fig. 5A) and that this also caused reduced growth rates when glutamate instead of ammonium was the sole nitrogen source for growth (Fig. 5B). This is a remarkable result, because it shows to our knowledge for the first time a physiological condition, essentially K^+^ limitation, where signaling through the PTS^Ntr^ phosphorylation cascade to PtsN dictates an essential cellular function, namely K^+^ homeostasis. And this is achieved through the strict regulation of two ATP dependent transporter classes. Under potassium limitation where potassium acquisition via the ATP dependent high affinity KdpABC system is essential to maintain intracellular potassium levels for growth, ATP dependent ABC transport is limited. This probably makes sense in a soil dwelling bacterium that is constitutively expressing a high number of ABC transport systems to acquire nutrients in an oligotrophic environment [7], [8]. Under K^+^ limitation such energy consuming efforts are shifted to K^+^ homeostasis as the primary need of the cell. In an enterobacterium such as *E. coli*, which probably rarely faces K^+^ limitation and facilitates transport primarily via PTS systems, PTS^Ntr^ regulation might have only evolved into modulatory roles, e.g. modulation of K^+^ uptake and PO_4_^3−^ acquisition.

## Materials and Methods

### Bacterial Growth and Media

The bacterial strains, plasmids and primers used in this study are detailed in Table 1. *Rhizobium* strains were grown at 28°C in either Tryptone Yeast extract (TY) [27] or acid minimal salts medium (AMS) [28] with 10 mM D-glucose as carbon source and 10 mM NH_4_Cl or 10 mM sodium glutamate as nitrogen sources. In cultures grown in AMS medium with defined potassium levels, K_2_HPO_4_ was replaced by Na_2_HPO_4_ and KCl added to appropriate concentrations. Antibiotics were used at the following concentrations ( µg ml^−1^): streptomycin (Str), 500; neomycin (Nm), 80; tetracycline (Tet), 5; gentamicin (Gm), 20; ampicillin (Amp) 100 and spectinomycin (Spec), 100.

**Table 1:** Strains, plasmids and primers.

Strains	Description	Reference, Source, Sequence
Rlv3841	*Rhizobium leguminosarum* bv *viciae*, Str^r^	[43]
PtsP107	Rlv3841 Tn5::*ptsP*	[10]
AA015	Rlv3841 *npr*::ΩTet	This study
AA006	Rlv3841 RL2904::ΩSpec	This study
AA016	Rlv3841 *npr*::ΩTet RL2904::ΩSpec	This study
AA031	Rlv3841 *npr*::ΩSpec	This study
RU4392	Rlv3841 *ask*:: ΩTet	[10]
Plasmids		
pJET1.2	Cloning vector	Thermo Fermentas
pJQ200SK	pACYC derivative; P15A origin of replication; Gm^r^	[44]
pRK415	IncP broad host range cloning vector; Tet^r^	[31]
pHMGWA	Expression vector; Amp^r^	[32]
pETduet-1	Expression vector; Amp^r^	Novagen Merck Millipore
pAA006	pJQ200SK carrying RL2904::ΩSpec	This study
pAA019	pJQ200SK carrying *npr*::ΩTet	This study
pAA037	pJQ200SK carrying *npr*::ΩSpec	This study
pLMB222	pHMGWA 6His-MBP-PtsP	This study
pAA033	pETduet 6His-NPr	This study
pLMB151	pRK415 × *ptsP*	[10]
pAA001	pRK415 × *ptsP*-GAF	This study
pAA003	pRK415 × *ptsP*H376A	This study
pAA038	pRK415 × *npr*	This study
pAA039	pRK415 × *npr*H17A	This study
Primer*		
P1nprforBam		TTTGGATCCTTTCAACGTCCATGCGACGG
P2nprrevXba		TTTTCTAGAAGCCTGCAATGCGCAGATCG
P3nprforinvSac		TTTGAGCTCATATAAACATATAAAGACT
P4nprrevinvSac		TTTGAGCTCAAGGCCCGTTATTTTCCGCT
P1RL2903forXho		TTTCTCGAGGAACCGGTGCTTGTGGCAGG
P2RL2903revXba		TTTTCTAGATCGAGCATGCGGATCGTTAC
P3RL2903forinvEco		TTTGATATCATGGCCGAACCGCTTAGGCT
P4RL2903revinvEco		TTTGATATCCAGGGGGACAGTTCCTCGGC
ptsPforHind		TTTAAGCTTTGATTCCCAGCGGGCGG
ptsPrevXba		TTTTCTAGATCGCCACCCTTCACTCCGAT
ptsP-GAFforSpe		TTTACTAGTCAGCGGCTCCGCCATCAACT
pstP-GAFrevSpe		TTTACTAGTGCCACCGGCGAGCTCAAGAA
ptsPH367Afor		AGGGGCGGTAACGAGCGCCGTGGTGATCGTTGCGC
ptsPH367Arev		GCGCAACGATCACCACGGCGCTCGTTACCGCCCCT
nprcompforHind		CCCAAGCTTGCGGAAAATAACGGGCCTTC
nprcomprevXba		CCCTCTAGATATATGAGCTCGATCGGAGC
nprH17Afor		AACAAGCGCGGTCTTGCCGCGCGCGCTTCCGCC
nprH17Arev		GGCGGAAGCGCGCGCGGCAAGACCGCGCTTGTT
ptsPexpfor		GGGGACAAGTTTGTACAAAAAAGCAGGCTTTATGAGAGACCTTTCCGGCGGTC
ptsPexprev		GGGGACCACTTTGTACAAGAAAGCTGGGTTCTAAAGGGGAATATTGTGGCTCTCGG
nprexpforBam		TTTGGATCCGACATCGCTCTCCCGGGAACT
nprexprevHind		TTTAAGCTTTCACATCTCTTCGCCAAACC

* Restriction sites in primer sequences are underlined.

### Mutant and Plasmid Construction

The *npr* (RL0032) mutant AA015 was isolated as follows. The *npr* gene was amplified with flanking DNA using primers P1nprforBam/P2nprrevXba (Tab. 1) and Phusion DNA polymerase (Finnzymes Thermo). The resulting PCR product was cloned into pJET1.2 (Fermentas Thermo) following the manufacturer’s instructions. An inverse PCR with primers P3nprforinvSac/P4nprrevinvSac was performed to amplify the vector lacking *npr*. The resulting PCR product was digested with *Ecl*136II and a *Sma*I digested ΩTet cassette [29] was inserted. From that construct a *Bam*HI/*Xba*I fragment was cloned into pJQ200SK to form pAA019. Strain AA015 (*npr*::ΩTet) was generated in the Rlv3841 wild type background by selecting for recombination using the sac mutagenesis strategy as previously described [30]. Strain AA015 was verified by PCR using primers P1nprforBam/P2nprrevXba confirming that the 2.3 kb wild type fragment was replaced by a 4 kb fragment. The *npr* mutant AA031 was isolated in the same way, but by inserting a *Sma*I digested ΩSpec cassette [29].

The RL2904 mutant AA006 was isolated as follows. The RL2904 gene was amplified with flanking DNA using primers P1RL2903forXho/P2RL2903frevXba and Phusion DNA polymerase. The resulting PCR product was cloned into pJET1.2 following the manufacturer’s instructions. An inverse PCR with primers P3RL2903forinvEco/P4RL2903revinvEco was performed to amplify the vector lacking RL2904. The resulting PCR product was digested with *Eco*RV and a *Sma*I digested ΩSpec cassette [29] was inserted. From that construct an *Xho*I/*Xba*I fragment was cloned into pJQ200SK to form pAA006. Strain AA006 (RL2903::ΩSpec) was generated in the Rlv3841 wild type background by selecting for recombination using the sac mutagenesis strategy as previously described [30]. Strain AA006 was verified by PCR using primers P1RL2903forXho/P2RL2903frevXba confirming that the 2.3 kb wild type fragment was replaced by a 4 kb fragment.

The *npr*::ΩTet RL2904::ΩSpec double mutant AA016 was generated using pAA019 (*npr*::ΩTet) and AA006 (RL2904::ΩSpec) as a recipient. Selection and verification was done as described above.

The complementing plasmids pAA001 and pAA003 were cloned in the following way. The *ptsP* ORF was amplified using primers ptsPforHind/ptsPrevXba and Phusion DNA polymerase. The resulting PCR product was cloned into pJET1.2 following the manufacturer’s instructions. An inverse PCR with primers ptsP-GAFforSpe/ptsP-GAFrevSpe was performed to amplify the vector lacking the *ptsP* GAF domain (aa 24–160). The resulting PCR product was digested with *Spe*I and religated. An *Xba*I/*Hin*dIII fragment was then cloned into pRK415 [31] forming pAA001. A second inverse PCR with primers ptsPH367Afor/ptsPH367Arev was performed and digested with *Dpn*I. After transformation a clone was identified by sequencing with the H367A mutation and an *Xba*I/*Hin*dIII fragment was then cloned into pRK415 forming pAA003.

The complementing plasmids pAA038 and pAA039 were cloned in the following way. The *npr* (RL0032) ORF was amplified using primers nprcompforHind/nprcomprevXba and Phusion DNA polymerase. The resulting PCR product was cloned into pJET1.2. An *Xba*I/*Hin*dIII fragment was then cloned into pRK415 forming pAA038. An inverse PCR with primers nprH17Afor/nprH17Arev was performed using pJET1.2 carrying *npr* as a template to generate the H17A mutant variant of *npr*. This was cloned as an *Xba*I/*Hin*dIII fragment into pRK415 forming pAA039.

The expression plasmids pLMB222 and pAA033 were cloned in the following way. The *ptsP* ORF excluding its stop codon was amplified using primers ptsPexpfor/ptsPexprev and Phusion DNA polymerase. The resulting PCR product was inserted into pHMGWA [32] using Gateway BP Clonase (Invitrogen) forming pLMB222. The *npr* ORF excluding its stop codon was amplified using primers nprexpforBam/nprexprevHind and Phusion DNA polymerase (Finnzymes). The resulting PCR product was cloned as a *Bam*HI/*Hin*dIII fragment into pETduet-1 (Novagen Merck Millipore) forming pAA033.

### Transport Assays

*R. leguminosarum* uptake assays were performed with 25 µM (4.625 kBq of ^14^C) solute [33], using cultures grown in AMS with 10 mM glucose and 10 mM NH_4_Cl to an OD_600_ of ∼0.4 (cultures with limiting K^+^ at ∼0.2). AMS medium with defined potassium levels were prepared as described above.

### Protein Purification

The expression plasmids pLMB222 (6His-MBP-PtsP) and pAA033 (6His-NPr) were electroporated into *E. coli* BL21 DE+ cells and LB Amp pre-cultures were grown overnight at 37°C. 500 ml LB Amp cultures were than inoculated and grown for ∼4 h at 37°C to an OD600 of 0.5–0.7. Those cultures were induced with 1 mM IPTG and grown over night at 18°C. Cells were harvested by centrifugation at 4°C, washed in 25 ml of PBS buffer and resuspended in 12.5 ml PBS. Cells were disrupted by sonication in extraction buffer (PBS; 0.1% Triton X-100; 10% glycerol; 5 mM β-mercaptoethanol) and extracts were cleared by centrifugation. Cell free extracts were loaded on Protino Ni-IDA 1000 (Macherey+Nagel, Germany) columns and the his-tagged proteins were purified following the manufacturer’s instructions (purification using native conditions protocol). The eluates were dialysed against the phosphorylation assay buffer (94 mM Tris HCl, pH8.0; 1.6 mM MgSO_4_; 10 mM β-mercaptoethanol) and protein purity was checked in an SDS PAGE following Coomassie blue staining (NuSep, Australia, precast gels, following manufacturer’s instructions).

### Phosphorylation Assay

[^33^P]PEP was synthesized in a 100 µl reaction mixture containing 0.1 M triethylamine (pH 7.6), 3 mM MgCl_2_, 15 mM KCl, 1 mM pyruvate, 0.1 mM PEP (cyclohexammonium salt), 10 µM [γ-^33^P]ATP (300 Ci/mmol), and 40 units of pyruvate kinase (Sigma) [34]. This assay only depends on the PEP/ATP ratio and transfers the vast majority of label from ATP to PEP. The reaction mixture was used as a [^33^P]PEP source without further purification. The phosphorylation assay was done following the protocol published by [26]. The assay was performed in 20 µl of assay buffer (see above), 2 µg of purified 6His-MBP-PtsP and/or 6His-NPr fusion protein and either 125 µM [^33^P]PEP (3 Ci/mmol) or 10 mM [γ-^33^P]ATP (3 Ci/mmol). Reactions were stopped in 6x Laemmli buffer and run on SDS PAGE. Radioactive labeled proteins were visualized on a Phosphoimager (Fujifilm FLA-3000). Gels were stained with Coomassie blue. RU4392 (Δask) crude extract was produced from 20 mL TY cultures by breaking the cells in a FastPrep FP120 instrument (Q-BioGen, USA) in phosphorylation assay buffer (see above). ∼10 µg of crude extract were used in the [γ-^33^P]ATP phosphorylation assay.

## References

[1] Oldroyd GE, Downie JA (2004) Calcium, kinases and nodulation signalling in legumes. Nat Rev Mol Cell Biol 5: 566–576. 15232574 10.1038/nrm1424

[2] Oldroyd GE, Downie JA (2008) Coordinating nodule morphogenesis with rhizobial infection in legumes. Annu Rev Plant Biol 59: 519–546. 18444906 10.1146/annurev.arplant.59.032607.092839

[3] Jones KM, Kobayashi H, Davies BW, Taga ME, Walker GC (2007) How rhizobial symbionts invade plants: the Sinorhizobium-Medicago model. Nat Rev Microbiol 5: 619–633. 17632573 10.1038/nrmicro1705 PMC2766523

[4] Oldroyd GE, Murray JD, Poole PS, Downie JA (2011) The rules of engagement in the legume-rhizobial symbiosis. Annu Rev Genet 45: 119–144. 21838550 10.1146/annurev-genet-110410-132549

[5] Prell J, Poole P (2006) Metabolic changes of rhizobia in legume nodules. Trends Microbiol 14: 161–168. 16520035 10.1016/j.tim.2006.02.005

[6] Mauchline TH, Fowler JE, East AK, Sartor AL, Zaheer R, et al (2006) Mapping the Sinorhizobium meliloti 1021 solute-binding protein-dependent transportome. Proc Natl Acad Sci U S A 103: 17933–17938. 17101990 10.1073/pnas.0606673103 PMC1635973

[7] Karunakaran R, Ramachandran VK, Seaman JC, East AK, Mouhsine B, et al (2009) Transcriptomic analysis of Rhizobium leguminosarum biovar viciae in symbiosis with host plants Pisum sativum and Vicia cracca. J Bacteriol 191: 4002–4014. 19376875 10.1128/JB.00165-09 PMC2698398

[8] Ramachandran VK, East AK, Karunakaran R, Downie JA, Poole PS (2011) Adaptation of Rhizobium leguminosarum to pea, alfalfa and sugar beet rhizospheres investigated by comparative transcriptomics. Genome Biol 12: R106. 22018401 10.1186/gb-2011-12-10-r106 PMC3333776

[9] Mulley G, White JP, Karunakaran R, Prell J, Bourdes A, et al (2011) Mutation of GOGAT prevents pea bacteroid formation and N2 fixation by globally downregulating transport of organic nitrogen sources. Mol Microbiol 80: 149–167. 21276099 10.1111/j.1365-2958.2011.07565.x

[10] Prell J, Mulley G, Haufe F, White JP, Williams A, et al (2012) The PTS(Ntr) system globally regulates ATP-dependent transporters in Rhizobium leguminosarum. Mol Microbiol 84: 117–129. 22340847 10.1111/j.1365-2958.2012.08014.x

[11] Deutscher J, Francke C, Postma PW (2006) How phosphotransferase system-related protein phosphorylation regulates carbohydrate metabolism in bacteria. Microbiol Mol Biol Rev 70: 939–1031. 17158705 10.1128/MMBR.00024-06 PMC1698508

[12] Reizer J, Reizer A, Saier MH Jr, Jacobson GR (1992) A proposed link between nitrogen and carbon metabolism involving protein phosphorylation in bacteria. Protein Sci 1: 722–726. 1304914 10.1002/pro.5560010604 PMC2142240

[13] Powell BS, Court DL, Inada T, Nakamura Y, Michotey V, et al (1995) Novel proteins of the phosphotransferase system encoded within the rpoN operon of Escherichia coli. Enzyme IIANtr affects growth on organic nitrogen and the conditional lethality of an erats mutant. J Biol Chem 270: 4822–4839. 7876255 10.1074/jbc.270.9.4822

[14] Reizer J, Reizer A, Merrick MJ, Plunkett G, 3rd, Rose DJ, et al (1996) Novel phosphotransferase-encoding genes revealed by analysis of the Escherichia coli genome: a chimeric gene encoding an Enzyme I homologue that possesses a putative sensory transduction domain. Gene 181: 103–108. 8973315 10.1016/s0378-1119(96)00481-7

[15] Cases I, Velazquez F, de Lorenzo V (2007) The ancestral role of the phosphoenolpyruvate-carbohydrate phosphotransferase system (PTS) as exposed by comparative genomics. Res Microbiol 158: 666–670. 17913467 10.1016/j.resmic.2007.08.002

[16] Young JP, Crossman LC, Johnston AW, Thomson NR, Ghazoui ZF, et al (2006) The genome of Rhizobium leguminosarum has recognizable core and accessory components. Genome Biol 7: R34. 16640791 10.1186/gb-2006-7-4-r34 PMC1557990

[17] Lee CR, Cho SH, Yoon MJ, Peterkofsky A, Seok YJ (2007) Escherichia coli enzyme IIANtr regulates the K+ transporter TrkA. Proc Natl Acad Sci U S A 104: 4124–4129. 17289841 10.1073/pnas.0609897104 PMC1794712

[18] Luttmann D, Heermann R, Zimmer B, Hillmann A, Rampp IS, et al (2009) Stimulation of the potassium sensor KdpD kinase activity by interaction with the phosphotransferase protein IIA(Ntr) in Escherichia coli. Mol Microbiol 72: 978–994. 19400808 10.1111/j.1365-2958.2009.06704.x

[19] Luttmann D, Gopel Y, Gorke B (2012) The phosphotransferase protein EIIA(Ntr) modulates the phosphate starvation response through interaction with histidine kinase PhoR in Escherichia coli. Mol Microbiol 86: 96–110. 22812494 10.1111/j.1365-2958.2012.08176.x

[20] Pfluger-Grau K, Gorke B (2010) Regulatory roles of the bacterial nitrogen-related phosphotransferase system. Trends Microbiol 18: 205–214. 20202847 10.1016/j.tim.2010.02.003

[21] Dozot M, Poncet S, Nicolas C, Copin R, Bouraoui H, et al.. (2010) Functional characterization of the incomplete phosphotransferase system (PTS) of the intracellular pathogen Brucella melitensis. PLoS One 5. 10.1371/journal.pone.0012679 PMC2937029 20844759

[22] Pfluger-Grau K, Chavarria M, de Lorenzo V (2011) The interplay of the EIIA(Ntr) component of the nitrogen-related phosphotransferase system (PTS(Ntr)) of Pseudomonas putida with pyruvate dehydrogenase. Biochim Biophys Acta 1810: 995–1005. 21236318 10.1016/j.bbagen.2011.01.002

[23] Pinedo CA, Bringhurst RM, Gage DJ (2008) Sinorhizobium meliloti mutants lacking phosphotransferase system enzyme HPr or EIIA are altered in diverse processes, including carbon metabolism, cobalt requirements, and succinoglycan production. J Bacteriol 190: 2947–2956. 18281401 10.1128/JB.01917-07 PMC2293241

[24] Pinedo CA, Gage DJ (2009) HPrK regulates succinate-mediated catabolite repression in the gram-negative symbiont Sinorhizobium meliloti. J Bacteriol 191: 298–309. 18931135 10.1128/JB.01115-08 PMC2612420

[25] Michiels J, Van Soom T, D’Hooghe I, Dombrecht B, Benhassine T, et al (1998) The Rhizobium etli rpoN locus: DNA sequence analysis and phenotypical characterization of rpoN, ptsN, and ptsA mutants. J Bacteriol 180: 1729–1740. 9537369 10.1128/jb.180.7.1729-1740.1998 PMC107084

[26] King ND, O’Brian MR (2001) Evidence for direct interaction between enzyme I(Ntr) and aspartokinase to regulate bacterial oligopeptide transport. J Biol Chem 276: 21311–21316. 11287431 10.1074/jbc.M101982200

[27] Beringer JE (1974) R factor transfer in Rhizobium leguminosarum. J Gen Microbiol 84: 188–198. 4612098 10.1099/00221287-84-1-188

[28] Poole PS, Schofield NA, Reid CJ, Drew EM, Walshaw DL (1994) Identification of chromosomal genes located downstream of dctD that affect the requirement for calcium and the lipopolysaccharide layer of Rhizobium leguminosarum. Microbiology 140 (Pt 10): 2797–2809. 10.1099/00221287-140-10-2797 8000544

[29] Fellay R, Frey J, Krisch H (1987) Interposon mutagenesis of soil and water bacteria: a family of DNA fragments designed for in vitro insertional mutagenesis of gram-negative bacteria. Gene 52: 147–154. 3038679 10.1016/0378-1119(87)90041-2

[30] Kumar S, Bourdes A, Poole P (2005) De novo alanine synthesis by bacteroids of Mesorhizobium loti is not required for nitrogen transfer in the determinate nodules of Lotus corniculatus. J Bacteriol 187: 5493–5495. 16030244 10.1128/JB.187.15.5493-5495.2005 PMC1196047

[31] Keen NT, Tamaki S, Kobayashi D, Trollinger D (1988) Improved broad-host-range plasmids for DNA cloning in gram-negative bacteria. Gene 70: 191–197. 2853689 10.1016/0378-1119(88)90117-5

[32] Busso D, Delagoutte-Busso B, Moras D (2005) Construction of a set Gateway-based destination vectors for high-throughput cloning and expression screening in Escherichia coli. Anal Biochem 343: 313–321. 15993367 10.1016/j.ab.2005.05.015

[33] Hosie AH, Allaway D, Galloway CS, Dunsby HA, Poole PS (2002) Rhizobium leguminosarum has a second general amino acid permease with unusually broad substrate specificity and high similarity to branched-chain amino acid transporters (Bra/LIV) of the ABC family. J Bacteriol 184: 4071–4080. 12107123 10.1128/JB.184.15.4071-4080.2002 PMC135202

[34] Roossien FF, Brink J, Robillard GT (1983) A simple procedure for the synthesis of [32P]phosphoenolpyruvate via the pyruvate kinase exchange reaction at equilibrium. Biochim Biophys Acta 760: 185–187. 6615882 10.1016/0304-4165(83)90141-1

[35] Boel G, Mijakovic I, Maze A, Poncet S, Taha MK, et al (2003) Transcription regulators potentially controlled by HPr kinase/phosphorylase in Gram-negative bacteria. J Mol Microbiol Biotechnol 5: 206–215. 12867744 10.1159/000071072

[36] Hu KY, Saier MH Jr (2002) Phylogeny of phosphoryl transfer proteins of the phosphoenolpyruvate-dependent sugar-transporting phosphotransferase system. Res Microbiol 153: 405–415. 12405346 10.1016/s0923-2508(02)01339-6

[37] Barabote RD, Saier MH Jr (2005) Comparative genomic analyses of the bacterial phosphotransferase system. Microbiol Mol Biol Rev 69: 608–634. 16339738 10.1128/MMBR.69.4.608-634.2005 PMC1306802

[38] Meyer FM, Jules M, Mehne FM, Le Coq D, Landmann JJ, et al (2011) Malate-mediated carbon catabolite repression in Bacillus subtilis involves the HPrK/CcpA pathway. J Bacteriol 193: 6939–6949. 22001508 10.1128/JB.06197-11 PMC3232832

[39] Becker A, Kuster H, Niehaus K, Puhler A (1995) Extension of the Rhizobium meliloti succinoglycan biosynthesis gene cluster: identification of the exsA gene encoding an ABC transporter protein, and the exsB gene which probably codes for a regulator of succinoglycan biosynthesis. Mol Gen Genet 249: 487–497. 8544814 10.1007/BF00290574

[40] Gorke B, Stulke J (2008) Carbon catabolite repression in bacteria: many ways to make the most out of nutrients. Nat Rev Microbiol 6: 613–624. 18628769 10.1038/nrmicro1932

[41] Bahr T, Luttmann D, Marz W, Rak B, Gorke B (2011) Insight into bacterial phosphotransferase system-mediated signaling by interspecies transplantation of a transcriptional regulator. J Bacteriol 193: 2013–2026. 21335451 10.1128/JB.01459-10 PMC3133047

[42] Rabus R, Reizer J, Paulsen I, Saier MH Jr (1999) Enzyme I(Ntr) from Escherichia coli. A novel enzyme of the phosphoenolpyruvate-dependent phosphotransferase system exhibiting strict specificity for its phosphoryl acceptor, NPr. J Biol Chem 274: 26185–26191. 10473571 10.1074/jbc.274.37.26185

[43] Johnston AW, Beringer JE (1975) Identification of the rhizobium strains in pea root nodules using genetic markers. J Gen Microbiol 87: 343–350. 1141859 10.1099/00221287-87-2-343

[44] Quandt J, Hynes MF (1993) Versatile suicide vectors which allow direct selection for gene replacement in gram-negative bacteria. Gene 127: 15–21. 8486283 10.1016/0378-1119(93)90611-6

